# Clinical outcomes of near-infrared photoimmunotherapy according to immune checkpoint inhibitor timing and treatment sequence: a multicenter retrospective study

**DOI:** 10.1007/s10147-026-03180-2

**Published:** 2026-09-01

**Authors:** Takuma Makino, Shohei Fujimoto, Yorihisa Orita, Tomoyasu Tachibana, Yuto Naoi, Mizuo Ando

**Affiliations:** 1 Department of Otolaryngology - Head and Neck Surgery, Graduate School of Medicine, Dentistry and Pharmaceutical Sciences, Okayama University, 2-5-1 Shikata-cho, Kita-ku, Okayama, 700-8558 Japan; 2 Department of Otolaryngology-Head and Neck Surgery, Graduate School of Medicine, Kumamoto University, Kumamoto, Japan; 3 Department of Otolaryngology Head and Neck Surgery, Japanese Red Cross Society Himeji Hospital, Himeji, Hyogo Japan

**Keywords:** Near-infrared photoimmunotherapy, Immune checkpoint inhibitor, Head and neck cancer, Treatment sequence, Local control

## Abstract

**Background:**

Near-infrared photoimmunotherapy (NIR-PIT) is a novel local treatment for recurrent or metastatic head and neck cancer. However, the clinical significance of immune checkpoint inhibitor (ICI) timing and treatment sequencing remains unclear. This study investigated their association with clinical outcomes after NIR-PIT.

**Methods:**

This multicenter retrospective study included 44 patients with 48 lesions who underwent initial NIR-PIT between January 2021 and March 2026. The primary analysis compared outcomes according to prior ICI exposure. An exploratory analysis evaluated outcomes according to treatment sequence and timing of ICI administration. The primary endpoint was local control. Secondary endpoints were objective response rate and disease control rate, while overall survival was assessed exploratorily.

**Results:**

Median local control duration was 253 days, but was 174 days in the prior ICI exposure group and 342 days in the non-prior ICI exposure group (log-rank *P* = 0.031). Local progression occurred in 69.2–37.1% of lesions, respectively (*P* = 0.059). Among 45 evaluable lesions, the objective response rate and disease control rate were 60.0–93.3%, respectively. Median overall survival was 866 days. Exploratory analyses suggested potential differences in clinical outcomes according to treatment sequence and ICI timing.

**Conclusions:**

Prior ICI exposure was associated with poorer local control after NIR-PIT. Exploratory analyses suggested that ICI timing and treatment sequencing may influence clinical outcomes. Treatment sequencing may represent an important consideration when integrating NIR-PIT and ICIs for recurrent or metastatic head and neck cancer.

## Introduction

Treatment for head and neck cancer (HNC) consists of multidisciplinary approaches combining surgery, radiotherapy, and systemic therapy according to tumor stage and primary site [1–3]. Despite these advances, a substantial proportion of patients develop recurrent or metastatic disease, and the management of recurrent/metastatic HNC remains a major clinical challenge [4]. In particular, treatment options for recurrent/metastatic disease have become increasingly diverse, depending on both prior treatments and patient condition.

Near-infrared photoimmunotherapy (NIR-PIT) has recently been introduced as a novel local treatment modality for recurrent/metastatic HNC, and favorable outcomes in local disease control and symptom palliation have been reported [5–7]. However, local recurrence and distant progression after NIR-PIT are not uncommon, and the selection of subsequent treatment remains an important clinical issue [8–10].

Immune checkpoint inhibitors (ICIs) are widely used in the management of recurrent or metastatic HNC, particularly for squamous cell carcinoma [11]. Recent studies have suggested that NIR-PIT may induce immunogenic cell death and modulate the tumor immune microenvironment [9, 12], leading to growing interest in combined or sequential use with ICIs. Further, concomitant use of NIR-PIT and ICIs has been reported as feasible and safe [13].

In real-world clinical practice, however, NIR-PIT and ICIs are not necessarily administered at the same time, and treatment sequences vary considerably among patients. For example, some patients have a history of ICI treatment before receiving NIR-PIT, whereas others receive ICIs shortly after NIR-PIT or as salvage therapy after disease progression following NIR-PIT [8]. Such differences in treatment sequence may influence local control and subsequent clinical outcomes after NIR-PIT. Nevertheless, most previous studies have focused on evaluating the efficacy of individual treatment modalities or on simple comparisons between treatments, and the relationship between ICI timing, treatment sequence, and clinical outcomes has not been adequately investigated. In particular, real-world evidence regarding the strategy of introducing ICIs before disease progression after NIR-PIT and the role of ICIs in patients who experience progression after NIR-PIT remains limited. Furthermore, the impact of ICI timing on local control and survival outcomes after NIR-PIT has yet to be fully elucidated.

We therefore primarily investigated the association between prior ICI exposure and clinical outcomes after NIR-PIT. We further performed exploratory analyses according to treatment sequence and timing of ICI administration.

## Materials and methods

### Study design and patients

This multicenter retrospective observational study investigated patients who underwent NIR-PIT for head and neck cancer. Okayama University Hospital served as the coordinating center, and the Department of Otolaryngology–Head and Neck Surgery at Kumamoto University Hospital and the Department of Otolaryngology at Japanese Red Cross Society Himeji Hospital participated in this study.

Eligible patients were those who underwent initial NIR-PIT for recurrent or metastatic HNC between January 2021 and March 2026. Data regarding patient characteristics, tumor-related factors, treatment details, clinical course, and outcomes were retrospectively collected from the medical records of each institution. Follow-up data were collected through June 2026.

### Group classification

For the primary analysis, patients were classified according to prior exposure to ICIs before NIR-PIT. Prior ICI exposure was defined as any history of ICI administration before the initial NIR-PIT session.

In an exploratory analysis, NIR-PIT-treated lesions were categorized according to treatment sequence and timing of ICI administration: G1, lesions with prior ICI exposure before NIR-PIT; G2, lesions treated with NIR-PIT alone; G3, lesions exposed to ICIs before disease progression after NIR-PIT; and G4, lesions exposed to ICIs after disease progression following NIR-PIT. The G3 group included lesions from patients who received ICIs as planned treatment or early sequential therapy after NIR-PIT. The G4 group included lesions from patients who started ICI therapy after confirmed progression of the NIR-PIT-targeted lesion. Disease progression for group classification was defined as radiologically confirmed progression of the NIR-PIT-targeted lesion.

### Data collection

The following variables were collected: age, sex, performance status (PS; ECOG), primary tumor site, presence of distant metastasis, date of initial NIR-PIT, number of NIR-PIT sessions, type of ICI, date of ICI initiation, tumor response, occurrence and date of local progression, date of subsequent treatment initiation, date of last follow-up, and survival status.

### Radiological assessment and treatment response

Tumor response was assessed based on radiological evaluations performed after NIR-PIT. Computed tomography or magnetic resonance imaging was used for imaging assessment, and treatment response was evaluated according to the Response Evaluation Criteria in Solid Tumors (RECIST) version 1.1 [14]. Radiological assessments, including both RECIST response evaluation and assessment of local progression, were performed by the treating investigators at each participating institution based on routine clinical imaging. No independent central radiological review was performed.

Tumor response was categorized as complete response (CR), partial response (PR), stable disease (SD), or progressive disease (PD). Best overall response (BOR) was defined as the best response recorded during the observation period. Objective response rate (ORR) was defined as the proportion of lesions achieving CR or PR, whereas disease control rate (DCR) was defined as the proportion achieving CR, PR, or SD.

ORR, DCR, and local control were evaluated on a per-lesion basis, whereas overall survival (OS) was evaluated on a per-patient basis.

### Endpoints

The primary endpoint was local control, which was defined as the interval between the date of initial NIR-PIT and the date of confirmed local progression. Local progression was defined as radiological enlargement or regrowth of the NIR-PIT-targeted lesion. Unlike RECIST-defined progressive disease, which reflects overall disease status, local progression was evaluated specifically for the NIR-PIT-targeted lesion because local control was the primary endpoint of this study. Consequently, progression at non-target lesions or distant metastatic sites according to RECIST was not considered a local progression event unless enlargement or regrowth of the NIR-PIT-targeted lesion was confirmed. Recurrence of the same lesion after achievement of CR was also considered local progression. Lesions without local progression were censored at the earlier of either the date of subsequent treatment initiation or the date of last follow-up. Subsequent treatment initiation was selected as a censoring event because additional therapies could directly influence local tumor status and confound assessment of local control attributable to NIR-PIT. Death was treated as a censoring event rather than a competing event. Secondary endpoints were ORR and DCR.

OS was analyzed as an exploratory endpoint. OS was defined as the interval between the date of initial NIR-PIT and the date of death from any cause. Patients who were alive as of the last follow-up were censored.

### Statistical analysis

Categorical variables were compared using the chi-square test or Fisher’s exact test, as appropriate. The same methods were used to compare local progression rate, ORR, and DCR between groups.

Local control and OS were estimated using Kaplan–Meier methods and compared using the log-rank test. Univariate Cox proportional hazards analysis was performed to explore the association between prior ICI exposure and local control.

All statistical tests were two-sided, with values of *P* < 0.05 considered significant. Because this was an exploratory retrospective study with a limited sample size and no clear candidate variables for multivariable analysis were evident from the univariate analyses, multivariable analysis was not performed. Median duration of follow-up was estimated using the reverse Kaplan–Meier method. All statistical analyses were performed using IBM SPSS Statistics for Windows, version 26.0 (IBM Corp., Armonk, NY, USA).

### Ethical considerations

All study protocols were approved by the institutional review board at Okayama University Graduate School of Medicine, Dentistry and Pharmaceutical Sciences, Japan (approval no. K2605-019). The study was conducted in accordance with the Declaration of Helsinki and institutional data-protection policies.

## Results

### Patient characteristics

**Fig. 1:**
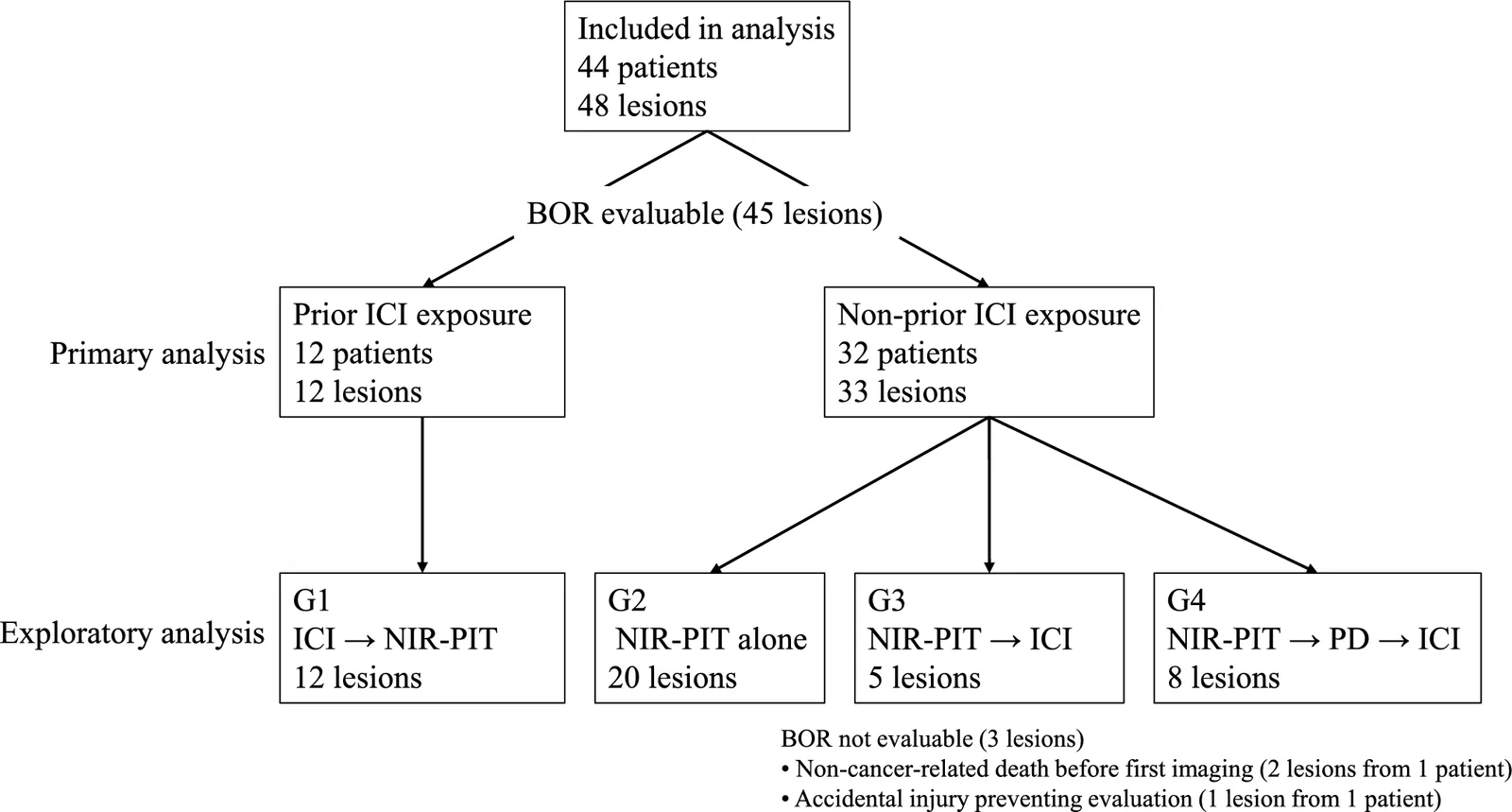
Study flow and group classification. Flow diagram of patient inclusion, lesion evaluation, and study group classification. Patients were classified according to prior immune checkpoint inhibitor (ICI) exposure for the primary analysis and according to treatment sequence and timing of ICI administration for the exploratory analysis

Participants comprised 44 patients with 48 lesions who underwent initial NIR-PIT for recurrent or metastatic HNC between January 2021 and March 2026. Median duration of follow-up as estimated using the reverse Kaplan–Meier method was 593 days. Twelve patients (27.3%) had prior ICI exposure and 32 patients (72.7%) had no prior ICI exposure. The patient selection process and study group classification are shown in Fig. 1.

Patient characteristics are summarized in Table 1. No significant differences were observed between the two groups with respect to age, sex, PS, primary tumor site, presence of distant metastasis, or number of NIR-PIT sessions. In contrast, a history of cetuximab treatment was significantly more frequent in the prior ICI exposure group (75.0%) than in the non-prior ICI exposure group (3.1%, *P* < 0.001).

**Table 1:** Baseline characteristics according to prior ICI exposure

Characteristic	Overall (n=44)	Prior ICI (n=12)	No prior ICI (n=32)	*p* value
Age, years	74 (65–78)	74 (62–78)	74 (66–78)	0.721
Male sex	31 (70.5)	9 (75.0)	22 (68.8)	1
ECOG PS ≥1	13 (29.5)	4 (33.3)	9 (28.1)	0.727
Primary site				0.224
Oral cavity	11 (25.0)	5 (41.7)	6 (18.8)	
Oropharynx	14 (31.8)	2 (16.7)	12 (37.5)	
Hypopharynx	4 (9.1)	2 (16.7)	2 (6.2)	
Larynx	4 (9.1)	0 (0.0)	4 (12.5)	
Sinonasal	4 (9.1)	1 (8.3)	3 (9.4)	
Nasopharynx	3 (6.8)	0 (0.0)	3 (9.4)	
Salivary gland	1 (2.3)	0 (0.0)	1 (3.1)	
Others	3 (6.8)	2 (16.7)	1 (3.1)	
Distant metastasis	5 (11.4)	2 (16.7)	3 (9.4)	0.603
Prior surgery	32 (72.7)	9 (75.0)	23 (71.9)	1
Prior radiotherapy	41 (93.2)	11 (91.7)	30 (93.8)	1
Prior irradiation to PIT site	39 (88.6)	10 (83.3)	29 (90.6)	0.603
Prior platinum therapy	31 (70.5)	11 (91.7)	20 (62.5)	0.075
Platinum-refractory disease	12 (27.3)	5 (41.7)	7 (21.9)	0.259
Prior cetuximab therapy	10 (22.7)	9 (75.0)	1 (3.1)	<0.001
PIT cycles	2 (1–3)	2 (1–3)	2 (1–2)	0.327

### Overall treatment outcomes

Overall treatment outcomes are summarized in Table 2. Among the 48 lesions, BOR was evaluable in 45 lesions. Three lesions were not evaluable because radiological assessment was unavailable before the initial response evaluation (two due to non-cancer-related death and one due to accidental injury). Among the 45 evaluable lesions, BOR was CR in 13 lesions, PR in 14 lesions, SD in 15 lesions, and PD in 3 lesions. ORR was 60.0% and DCR was 93.3%.

**Table 2:** Overall outcomes of NIR-PIT

Outcome	Value
Patients	44
Treated lesions	48
BOR evaluable lesions	45
CR	13
PR	14
SD	15
PD	3
ORR	27/45 (60.0%)
DCR	42/45 (93.3%)

Three lesions were not evaluable for BOR because radiological assessment was unavailable before the initial response evaluation (two due to non-cancer-related death and one due to accidental injury)

**Fig. 2:**
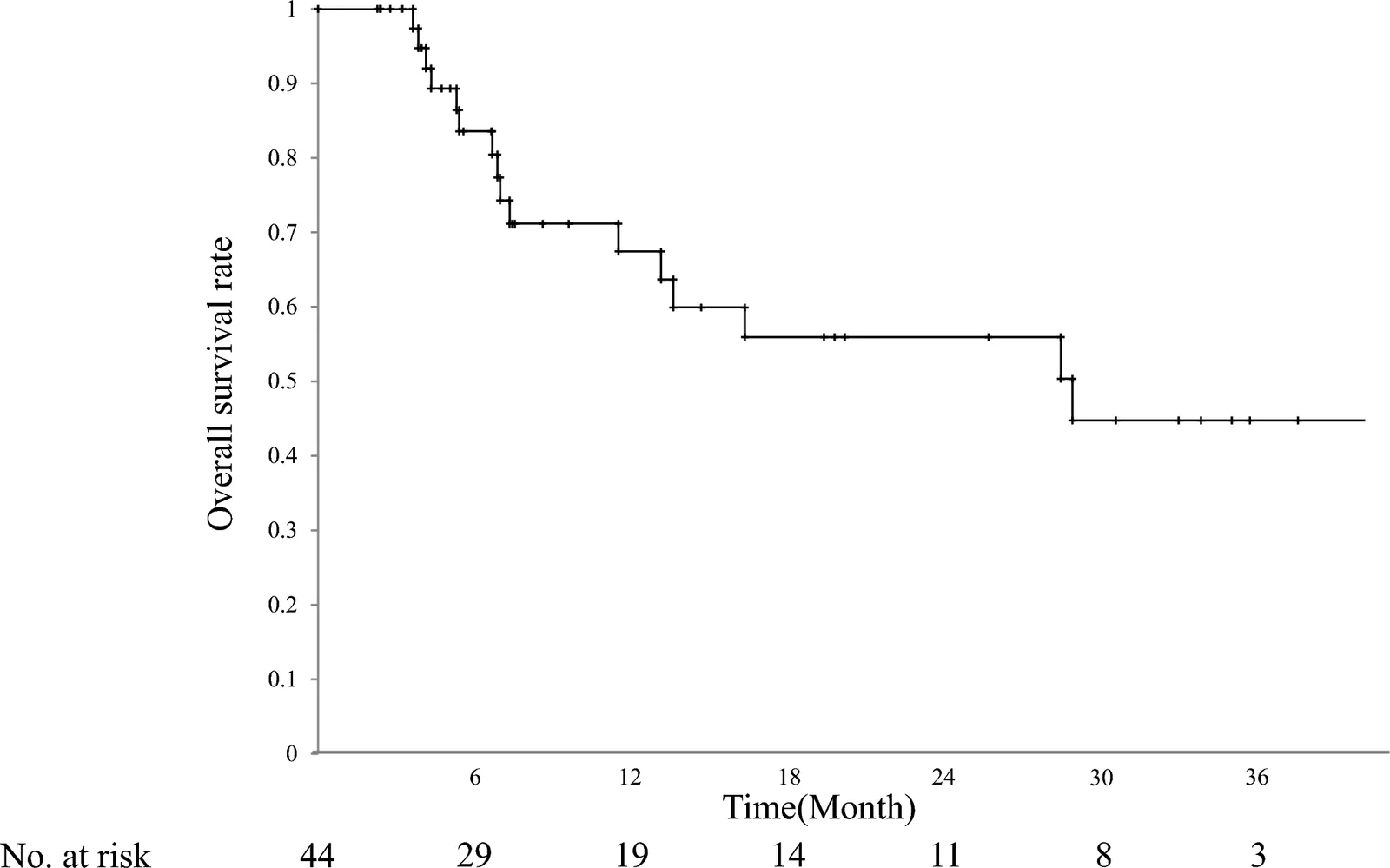
Overall survival after NIR-PIT. Kaplan–Meier curve for overall survival (OS) in the entire cohort. Median OS was 866 days. Six-, 12-, and 24-month OS rates were 83.5%, 67.4%, and 55.9%, respectively

During the observation period, 17 patients died. Median OS was 866 days. Six-, 12-, and 24-month OS rates were 83.5%, 67.4–55.9%, respectively (Fig. 2).

**Fig. 3:**
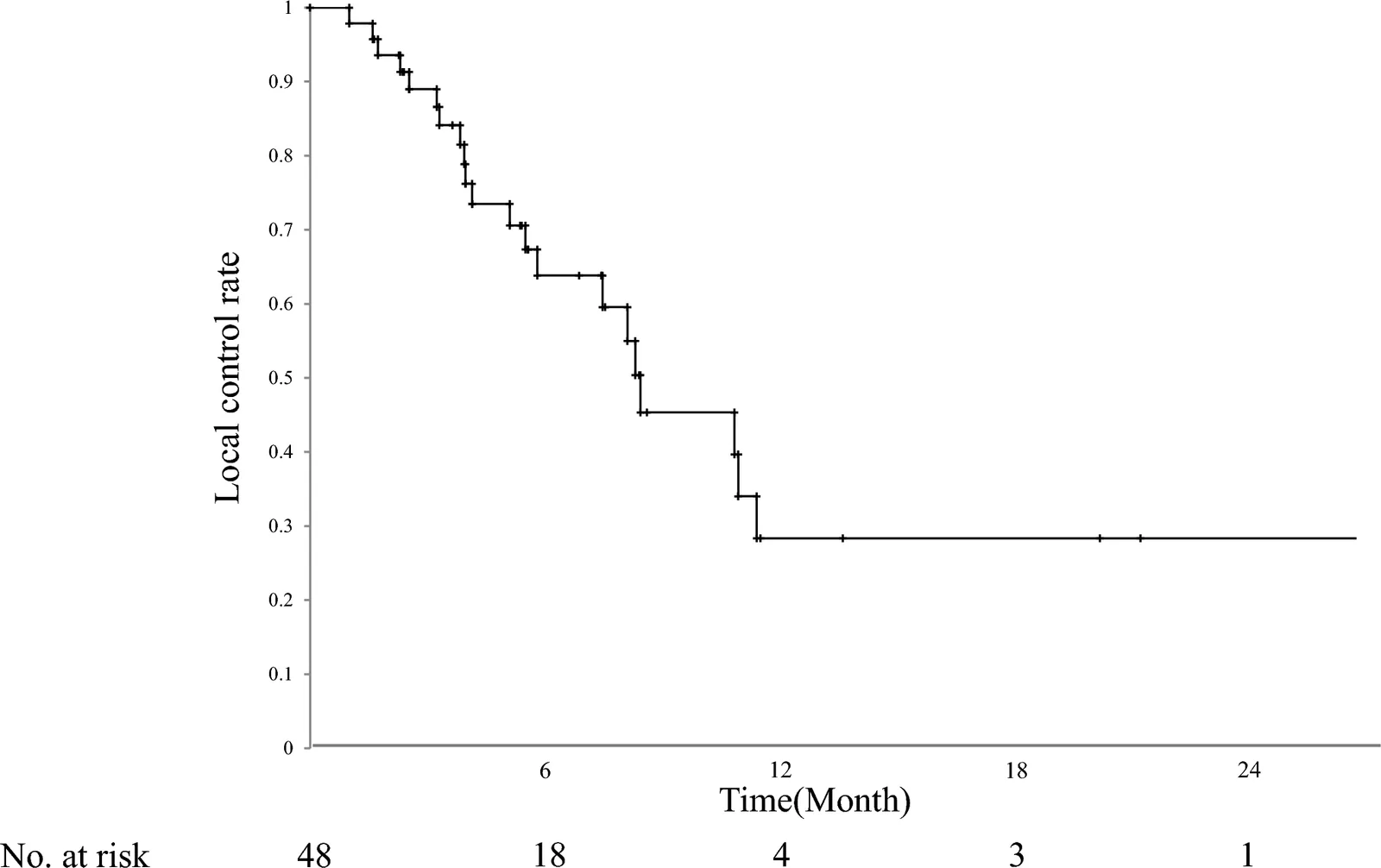
Local control after NIR-PIT. Kaplan–Meier curve for local control in the entire cohort. The median local control duration was 253 days. Six- and 12-month local control rates were 63.8–28.3%, respectively

Local progression was identified in 22 of the 48 lesions (45.8%). Median local control duration was 253 days, and 6- and 12-month local control rates were 63.8–28.3%, respectively (Fig. 3).

### Treatment outcomes according to prior ICI exposure

Treatment outcomes according to prior ICI exposure are shown in Table 3. BOR-based ORRs were 75.0% in the prior ICI exposure group and 54.5% in the non-prior ICI exposure group. Corresponding DCRs were 91.7–93.9%, respectively. No significant differences in either rate were observed between groups.

**Table 3:** Outcomes according to prior ICI exposure

Outcome	Prior ICI	No prior ICI	*p* value
ORR	9/12 (75.0%)	18/33 (54.5%)	0.308
DCR	11/12 (91.7%)	31/33 (93.9%)	1
Local progression	9/13 (69.2%)	13/35 (37.1%)	0.059

BOR was evaluable in 45 lesions (12 and 33 lesions, respectively). Local progression was evaluated in all 48 lesions

Local progression was observed in 9 of 13 lesions (69.2%) in the prior ICI exposure group and in 13 of 35 lesions (37.1%) in the non-prior ICI exposure group. Although the local progression rate tended to be higher in the prior ICI exposure group, this difference did not reach the level of statistical significance (Fisher’s exact test, *P* = 0.059).

### Local control according to prior ICI exposure

**Fig. 4:**
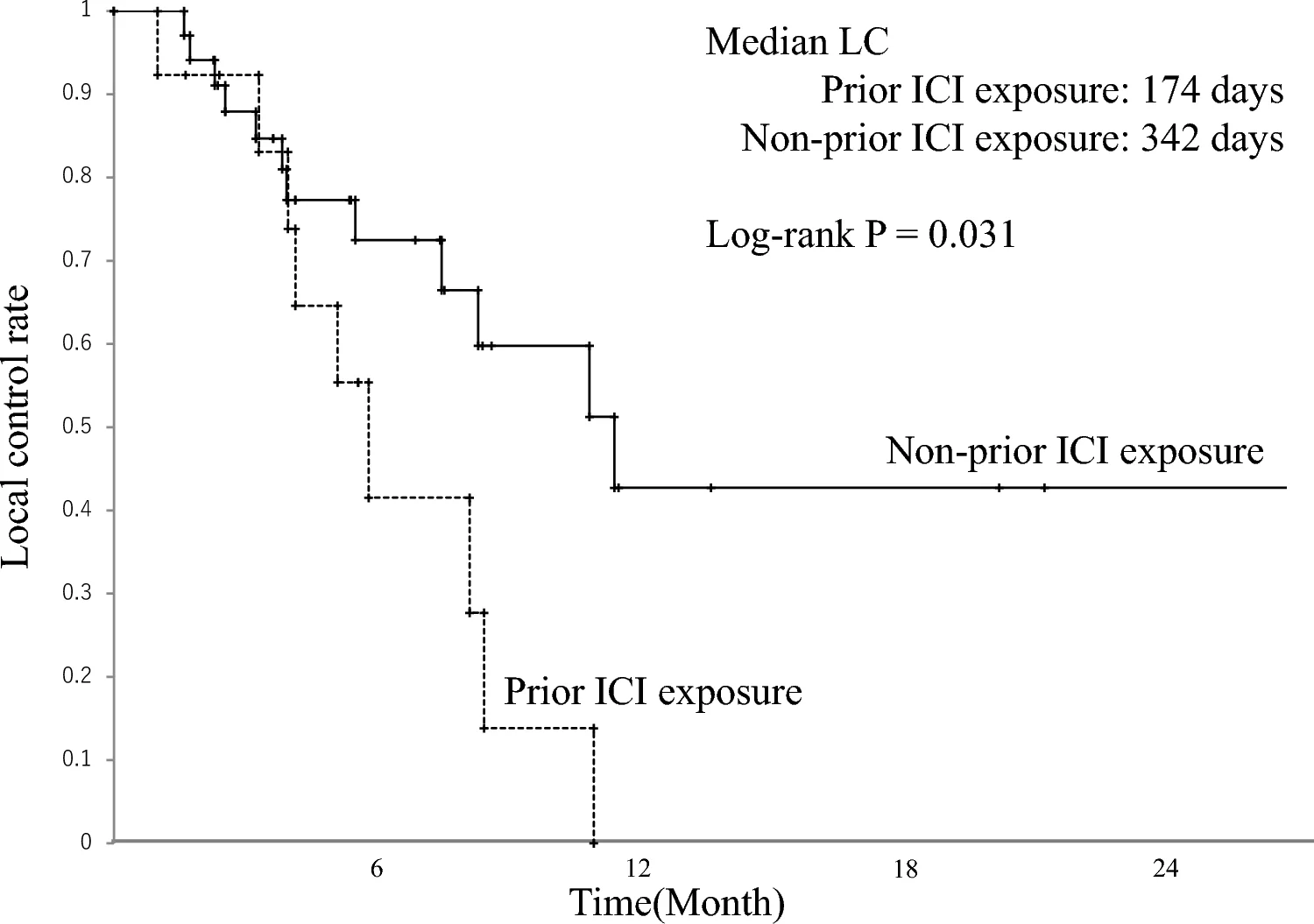
Local control according to prior immune checkpoint inhibitor exposure. Kaplan–Meier curves for local control according to prior immune checkpoint inhibitor (ICI) exposure before NIR-PIT. Median local control durations were 174–342 days in the prior and non-prior ICI exposure groups, respectively. Local control was significantly poorer in the prior ICI exposure group (log-rank *p* = 0.031)

Kaplan–Meier curves for local control are shown in Fig. 4. Median duration of local control was 174 days in the prior ICI exposure group and 342 days in the non-prior ICI exposure group. Kaplan–Meier analysis demonstrated significantly poorer local control in the prior ICI exposure group (log-rank *P* = 0.031).

In the univariate Cox proportional hazards analysis, prior ICI exposure tended to be associated with an increased risk of local progression (hazard ratio, 2.38; 95% confidence interval, 1.00–5.70; *P* = 0.051).

### Exploratory analysis according to treatment sequence and ICI timing

The results of the exploratory analysis according to treatment sequence and ICI timing are summarized in Table 4. BOR-based ORRs were 75.0% in G1, 65.0% in G2, 40.0% in G3, and 37.5% in G4. Corresponding DCRs were 91.7%, 95.0%, 100%, and 87.5%, respectively. Local progression was observed in 69.2% of lesions in G1, 22.7% in G2, 40.0% in G3, and 75.0% in G4. These findings suggest potential differences in treatment outcomes according to treatment sequence, although interpretation should be cautious because of the limited sample size.

**Table 4:** Exploratory outcomes according to treatment sequence and timing of ICI administration

Sequence	Definition	Lesions	BOR (CR/PR/SD/PD)	ORR	DCR	Local progression
G1	ICI before NIR-PIT	13	5/4/2/1	9/12 (75.0%)	11/12 (91.7%)	9/13 (69.2%)
G2	NIR-PIT alone	22	7/6/6/1	13/20 (65.0%)	19/20 (95.0%)	5/22 (22.7%)
G3	ICI after NIR-PIT before progression	5	1/1/3/0	2/5 (40.0%)	5/5 (100.0%)	2/5 (40.0%)
G4	ICI after progression following NIR-PIT	8	0/3/4/1	3/8 (37.5%)	7/8 (87.5%)	6/8 (75.0%)

BOR was evaluable in 45 lesions (12, 20, 5, and 8 lesions in G1–G4, respectively)

Local progression was evaluated in all 48 lesions (13, 22, 5, and 8 lesions in G1–G4, respectively)

## Discussion

This study explored the associations between ICI timing, treatment sequence, and clinical outcomes among patients treated with NIR-PIT. We found that patients with prior exposure to ICI demonstrated poorer local control and higher rates of local progression. In addition, exploratory analyses suggested potential differences in clinical outcomes according to treatment sequence and timing of ICI administration. Collectively, these findings suggest that the timing of ICI administration and treatment sequence before and after NIR-PIT may be associated with treatment outcomes. Overall survival outcomes in the present cohort were generally consistent with those reported in previous real-world clinical studies of NIR-PIT, supporting the representativeness of the study population [15, 16].

The most important finding of the present study was the poorer local control observed in patients with prior ICI exposure. However, this finding does not necessarily indicate that prior ICI treatment itself reduces the efficacy of NIR-PIT. The prior ICI exposure group included patients who had experienced disease progression despite ICI treatment, and such patients may harbor tumors with immunological treatment resistance [17, 18]. The poorer local control observed in this group may thus reflect selection of patients with more treatment-resistant disease rather than a direct effect of prior ICI exposure itself. Indeed, the local progression rate was 69.2% in the prior ICI exposure group compared with 37.1% in the non-prior ICI exposure group. Although this difference did not reach statistical significance, the observed trend was consistent with the poorer local control demonstrated by Kaplan–Meier analysis and warrants further investigation in larger cohorts. Furthermore, local control duration was significantly shorter in the prior ICI exposure group, suggesting potential differences in tumor biology at the time of NIR-PIT.

Although NIR-PIT is primarily a local treatment modality, accumulating preclinical evidence suggests that the effects extend beyond direct tumor cell destruction and may involve the induction of antitumor immune responses through immunogenic cell death [19]. Consequently, durable tumor control after NIR-PIT may depend not only on local cytotoxic effects, but also on host immune responses. From this perspective, the poorer local control observed with the prior ICI exposure group is particularly noteworthy. Tumors that progress despite ICI treatment may exhibit an immune-evasive phenotype characterized by T-cell dysfunction, an immunosuppressive tumor microenvironment, or acquired mechanisms of immune escape [20]. As previously suggested [12], even when NIR-PIT induces tumor cell destruction in such tumors, subsequent antitumor immune responses may not be sufficiently activated, potentially resulting in local recurrence or regrowth. Although the present study did not include immunological analyses and therefore cannot directly validate this hypothesis, the consistently poorer local control observed among patients with prior ICI exposure may reflect an underlying treatment-resistant biological state rather than differences in treatment history alone.

In the exploratory analysis, poorer local control was observed in both patients with prior ICI exposure before NIR-PIT (G1) and those who received ICIs after disease progression following NIR-PIT (G4), whereas patients who received ICIs before disease progression after NIR-PIT (G3) demonstrated durable disease stabilization despite relatively modest response rates. Although the small sample size precludes definitive conclusions, these observations suggest that differences in treatment sequence may be associated with clinical outcomes. Recent studies have suggested that NIR-PIT can induce antitumor immune responses through immunogenic cell death and may also remodel the tumor immune microenvironment by altering CD8+ T-cell distribution and immune-cell PD-L1 expression [16, 21]. Therefore, administration of ICIs during the early immunological changes induced by NIR-PIT may enhance antitumor immunity. Preclinical studies have further demonstrated that immune-cell-targeted NIR-PIT can enhance antitumor immunity through depletion of immunosuppressive cell populations such as regulatory T cells and VISTA-expressing immune cells, resulting in increased CD8+ T-cell activation and a more favorable CD8+/Treg balance [22, 23]. These findings suggest that the immunologically active period immediately after NIR-PIT may represent a window during which immune checkpoint blockade could more effectively augment antitumor immunity. In the present study, lesions receiving ICIs before documented progression after NIR-PIT (G3) showed durable disease stabilization, whereas lesions treated with ICIs only after progression (G4) demonstrated less favorable local control. Although these observations are exploratory and should be interpreted cautiously because of the limited sample size, they are biologically consistent with the hypothesis that earlier introduction of ICIs may better exploit NIR-PIT-induced immune activation. While the optimal treatment sequence remains undefined, the favorable disease stabilization observed in patients who received ICIs before documented progression after NIR-PIT (G3) suggests that earlier introduction of ICIs during the period of NIR-PIT-induced immune activation may represent a promising treatment strategy. Because this observation was derived from a small exploratory cohort, prospective studies are warranted to validate this hypothesis.

Several limitations of this study should be acknowledged. First, this was a retrospective observational study and was therefore subject to selection bias and unmeasured confounding. In particular, many patients in the prior ICI exposure group had experienced disease progression despite prior ICI treatment. This raises the possibility of selection bias, as these patients may have represented a cohort with inherently more treatment-resistant tumor biology. Therefore, the poorer local control observed in this group may reflect a more treatment-resistant biological phenotype rather than a direct effect of prior ICI exposure itself. The marked imbalance in prior cetuximab exposure between the two groups also represents a potential source of confounding. Patients in the prior ICI exposure group were more likely to have received prior cetuximab therapy, reflecting a heavier pretreatment burden and more complex treatment history. Therefore, the poorer local control observed in the prior ICI exposure group may reflect differences in treatment history and underlying tumor biology rather than the effect of prior ICI exposure alone. Because of the limited sample size and retrospective design, the independent contribution of prior cetuximab exposure could not be determined in the present study. Further, local control was evaluated on a per-lesion basis, and statistical independence among lesions could not be fully guaranteed. Radiological assessments were performed by investigators at each participating institution without independent central review, which may have introduced inter-observer variability and assessment bias. In addition, the interval between the last ICI administration and NIR-PIT was not evaluated because the primary objective was to investigate treatment sequencing in a real-world setting rather than the pharmacological carry-over effects of ICIs. Nevertheless, this factor may have influenced the observed outcomes. Second, the sample size was limited, particularly in the exploratory analysis of treatment sequence, markedly limiting statistical power and precluding reliable multivariable analysis for potential confounding factors. Therefore, the findings should be interpreted cautiously. In addition, because the median follow-up duration was shorter than 24 months, the estimated 24-month overall survival rate should be interpreted with appropriate caution because it was based on a limited number of patients remaining at risk. Third, immunological and tumor microenvironment analyses were not performed, so the biological mechanisms underlying the observed differences in clinical outcomes, including the proposed hypothesis of T-cell dysfunction, could not be investigated. Consequently, the proposed explanations regarding treatment resistance and treatment sequence should be considered hypothesis-generating. Nevertheless, this study is clinically relevant because it focuses on treatment sequence in real-world NIR-PIT and ICI practice and suggests a potential association between ICI timing and clinical outcomes. Future large-scale prospective and translational studies are warranted to validate these exploratory findings and to determine the optimal treatment sequence for integrating NIR-PIT and ICIs.

## Conclusion

Prior ICI exposure was associated with poorer local control after NIR-PIT. In addition, exploratory analyses suggested that the treatment sequence of NIR-PIT and ICIs, as well as the timing of ICI administration, may be associated with clinical outcomes. These findings indicate that treatment sequencing represents an important consideration in integrated therapeutic strategies combining NIR-PIT and ICIs for recurrent or metastatic HNC. Further validation of the clinical significance and underlying biological mechanisms through prospective and translational studies is warranted.

## Data Availability

The datasets generated and/or analyzed during the current study are available from the corresponding author on reasonable request.
